# A Qualitative Study on Perceptions of Changes Reported by Caregivers of Patients in Vegetative State and Minimally Conscious State: The “Time Gap Experience”

**DOI:** 10.1155/2014/657321

**Published:** 2014-11-06

**Authors:** Venusia Covelli, Milda Cerniauskaite, Matilde Leonardi, Davide Sattin, Alberto Raggi, Ambra Mara Giovannetti

**Affiliations:** Neurological Institute Carlo Besta IRCCS Foundation, Neurology, Public Health and Disability Unit, Via Celoria 11, 20133 Milan, Italy

## Abstract

*Objective*. Our purpose was to provide a comprehensive understanding of how women informal caregivers of patients in vegetative state (VS) or minimally conscious state (MCS) describe, represent, and experience changes that occurred in their life after the acute event of their family member. *Methods*. A qualitative study was conducted and fifteen women informal caregivers, mothers, or spouses of patients in VS or MCS were interviewed. *Results*. Caregivers' narratives revealed (1) important personal and interpersonal changes and (2) difficulties while facing the complex situation and integrating past, present, and future, defined as a “time gap experience.” This difficulty is expressed in two ways. First, the reduction of variety of roles into one, caregiver's role. Second, the relationship with the relative is characterised by fluctuation in the relational style between caregiver and relative; it shifts from an adult to adult interaction to an adult to child one. Another fluctuation can be observed in the mixed use of present and past tenses when caregivers speak about their relatives. *Conclusions*. Caregiving cause pervasive modifications in one's life. Targeted interventions aiming to empower the caregivers, to support them after the acute event in caregiving activities together with patient-focused interventions, and to promote their health should be implemented.

## 1. Introduction

Vegetative state (VS) and minimally conscious state (MCS) are both clinical conditions acquired or developed following severe brain injury, also there are clinically classified as disorders of consciousness (DOC). Patients in VS are in a state of wakefulness, but they completely fail to show any sign of awareness of themselves and of the environment [1], while MCS is a condition in which minimal but reproducible and finalistic behavioural evidence of awareness is demonstrated [2]. Some clinicians suggest using “unresponsive wakefulness syndrome” (UWS) definition instead of “vegetative state” to reduce the negative social stigma when referring to patients as “being vegetable-like” [3].

Because of their severe clinical condition, people in VS or MCS require demanding and constant assistance from formal and informal caregivers [4–6]. Formal caregivers are health workers paid or trained by statutory bodies and, due to the management of heavy health clinical condition of patients in VS or MCS, they might experience high level of burnout [7, 8]. Informal caregivers, instead, are persons who care for a friend, family member, or neighbor and whose care is not based on any formal agreement or services specifications [9]. About VS and MCS patients, informal caregivers are usually next of kin women [10, 11] (mother, spouse, or partner); married; employed, with a mean age of 52 years [12]. Studies show that these caregivers spend a lot of time taking care of their relative, even if they work. They report an abrupt modification of their life, consistent reduction of work duties, interests and hobbies, and social interactions [13]. Life expectancy of patients in VS or MCS is estimated to be over ten years [14, 15] and in some cases patients live more than 20 years [16]. This implies long-term caring and changes in daily life of caregivers for many years.

It has been shown that caregivers of patients in VS and MCS experience high levels of physical, emotional, social, and financial burden and generally report worse health than general population [17]. Despite clinical differences in conditions of VS and MCS, there are no differences in perceived caregivers' burden and distress between them [12]. Moreover, a study showed low economic profile of caregivers and poor physical and mental health as well as severe emotional burden, expressed in high levels of depressive and anxiety symptoms, decrease in leisure time, and continuous request for information from health professionals [17]. A recent study also show that the relation between depressive and anxiety symptoms, perceived burden and needs expressed is mediated by burden, where higher burden accentuates and lower burden mitigates the needs expressed by caregivers [18].

Previous research that studied attitudes and reactions of caregivers and family members towards patients in VS obtained similar findings. Results highlighted a reduction of personal interests, unsatisfactory family relationships, economic problems, and restrictions in social relationships [13, 19, 20]. High psychological burden, defined as mental health status and reported level of anxiety and depressive symptoms, was found in the study of caregivers of children in VS and MCS too [21]. Authors [22] have also found that caregivers' perceptions about the patient in VS' awareness of pain, light or darkness, taste, and verbal expression are related to what family members think about the pace and treatment of relative's clinical condition; see also [23, 24]. More recently, a qualitative study from Israel addressed the meaning of being the wife of a patient in VS and reported two parallel and contrasting experiences: one characterized by an enhanced self-esteem and inner strength and the other filled with emotional grief, a sense of isolation, and diminished hope about the future [25].

Studies that address various issues related to being a caregiver, its consequences on daily life, on perception of a relative-patient, on role definition, and identity changes are mainly conducted with caregivers of terminally ill or chronically ill patients. Women caregivers of stroke survivors, for example, described their experience as losing the life that once was, characterised by feeling of being overwhelmed, missing personal time, and facing uncertain future [26]. Due to the changes related to the relatives' health condition, stroke patients no longer seemed the same persons that the caregiver had known [27, 28]. Moreover, seventeen caregivers of persons with advanced cancer were interviewed about their role and the impact of caregiving on their lives. Male and female caregivers' narratives indicated a common difficulty in defining their activities of taking care of the patient as a distinct role, and they describe their daily work as general support to the relative. Some caregivers also experienced a loss of self-identity and had difficulties in taking a break or receiving help [29].

Furthermore, a modified image of self was described by caregivers who had a dying family member at home. They reported reduced space for intimacy and privacy, as well as “interdependency” that was expressed in decreased autonomy of caregiver because the rhythm of his/her daily life was very dependent on the relative's rhythm [30]. Similar findings came from a study on informal caregivers of patients at the end of life, where they reported a lack of identification with the caregiver's role [31]. Loss of identity and the need to maintain their own sense of self were also described by caregivers of patients with different diagnoses, such as Alzheimer's, Parkinson's, heart diseases, and cancer [32]. Sensation of losing oneself was previously reported by family members of patients with Alzheimer's disease. For spouses, women, and younger caregivers it was often a result of engulfment in the caregiver's role [33].

Few studies specifically address how women informal caregivers of patients in VS or MCS perceive their life after the acute event. To the best of our knowledge, no previous studies have been done comparing different informal caregivers (spouses/partners or mothers) and the caregiving for patients with different diagnosis (VS or MCS), place where they are hosted (at home, postacute rehabilitation care, long-term care) and duration of health condition. Therefore, the central question of our paper is twofold: (1) to give a better understanding of caregivers' experience while taking into account caregivers and patients with different features and (2) to develop an explanatory theory that might emerge from the collected data. Results might contribute to a greater awareness of health professionals, eventually leading to the development of interventions to support these caregivers.

## 2. Materials and Methods

We attempt to investigate how women informal caregivers of patients in VS or MCS perceive changes in their life after the acute event. Thus, a qualitative methodology was chosen and thematic analysis was realised to examine the data collected. Our research did not start from a specific concept to test how to explain caregivers' perceptions but arose from the challenge to find a theory emerging from data collected. For this reason, we adopted a grounded theory approach that provides the most suitable method [34].

For a period of six months while their relative was at the Coma Research Centre (CRC) of the Neurological Institute Carlo Besta IRCCS Foundation in Milan, women informal caregivers were recruited and interviewed. In particular, the CRC provides a 1-week programme of clinical, neurological, neurophysiological, and neuroradiological assessment for diagnosis and prognosis of patients with DOC. Coherently with results of previous studies that showed that caregivers of patients in VS and MCS are mainly women, only women informal caregivers were selected [10, 12, 17]. Our research received the approval from the Centre's ethics committee. Caregivers participated in one in-depth interview with a psychologist researcher (VC or AMG) and provided informed consent so that confidentiality was preserved. Interviews were conducted in person in a private room in the hospital and recruitment continued until saturation of themes was achieved [34]. All the interviews were recorded and they lasted for an average of 43 minutes (range: 32–64 min).

The interviews began with a brief sociodemographic form to collect data on age, relationship with the patient, data of time from acute event, and place where the patient lives. The interview continued with a general question about changes in caregivers' life after the acute event: Would you describe your experience or what did change after the acute event? During the interview, the researcher used additional prompts to help caregivers elaborate their answers and favour an in-depth description of the experience lived, with particular attention to self-perception and perception about the relative.

Preliminary analysis on demographic data was done using frequencies and descriptive statistics to describe the participants. Once the interviews were transcribed verbatim, QRS NVivo 10.0 [35] was used to organize and analyse the material into themes. We proceeded with a thematic analysis of data collected without a preconceived coding scheme. The analysis was composed of different steps. Each interview was read and reread by three researchers (Venusia Covelli, Milda Cerniauskaite, and Ambra Mara Giovannetti) to get familiar with material and to start identifying emerging subthemes and themes. After the first twelve interviews were done, no new themes emerged and the saturation was reached for the main themes identified. The saturation was confirmed with three subsequent interviews and no more interviews were conducted [36]. When all fifteen interviews were completed, the whole set of transcribed interviews was read and reread to define the final themes and complete the coding process. Disagreements among judges were resolved through discussion until consensus was reached.

Results will be presented citing mothers and partners. When relevant, differences for place of living and time from acute event will be specified. Every citation will be followed by a brief code in brackets, for example, 4-M-HOME-7,4y that identifies number of interview, relationship of caregiver with a relative (M = mother; P = partner), place where the relative is hosted (HOME = at home; INST = long-term care institution), and time from the acute event (expressed in years). Names of relatives in the citations were changed in order to preserve their privacy.

## 3. Results

Fifteen women informal caregivers were interviewed. Mean age was 57 (range: 32–78) and mean duration of relative's health condition was 5 years (range: 2–17). Seven caregivers were spouses/partners and eight mothers. Seven caregivers had their relative in a long-term care centre while the remaining patients lived at home with their caregivers. Seven caregivers had a relative in VS and eight in MCS. Demographic data of caregivers and patients are presented in Table 1.

**Figure 1:**
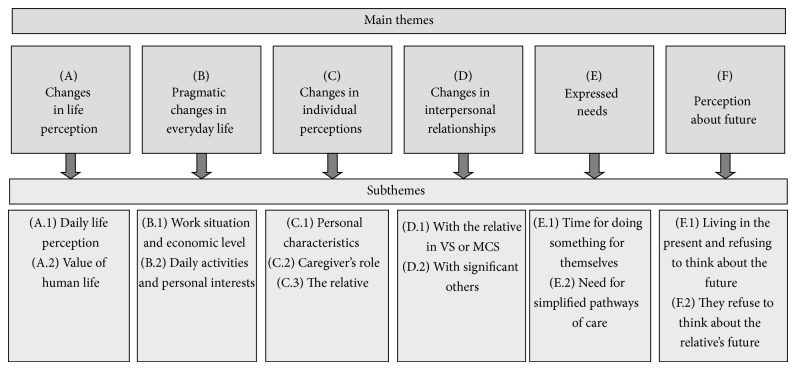


The analysis of the entire corpus of interviews allowed us to detect the main themes in caregivers' perceptions about changes in their life after the acute event. Six main themes emerged and a summary of each one is provided below. A graphic representation of themes and subthemes outlined is presented in Figure 1.

The six main themes that emerged do not differ between mothers or partners with a relative in VS or in MCS, while differences for place of living (at home versus long-term care institutions) and time from acute event emerged. Moreover, caregivers' education level and the kind of care provided by caregivers (e.g., toileting, caring for teeth and hair, and tracheal suctioning, providing nutrition and liquids) do not influence our results.  Table 2 shows details of caregivers listed in order in which they were interviewed.

### 3.1. Changes in Life Perception

After the prompt generic question about what changed after the acute event, caregivers reported general considerations about perceptions on their life and the value of human life.

#### 3.1.1. Daily Life Perception

The first response to the question about what changed after the acute event, expressed by almost all caregivers, was a total modification of their life. A mother reported the following: “I was overwhelmed, certainly there is the anxiety to find out your life suddenly and completely changed, and your son in this condition. You don't even think about it, that's it” (1-M-HOME-11y). A partner said as well the following: “Now I am about to cry. What should I say? What has changed? Everything. I don't care about my home anymore, I'm leaving aside everything” [8-P-INST-1y].

#### 3.1.2. Value of Human Life

A mother said the following: “Respect for life has changed, respect for every form of life because, you know, you now understand how it is important to respect people like Luigi, people that sometimes are not respected, they could be abused” [2-M-HOME-2,7y]. A wife said the following: “First of all, I have understood a lot of things, for example I now understand what's love. I have realized that often we have a distorted idea of love. We think that we love someone but instead we love the fact that the other person makes us feel good, or gives us things we expect to receive.” [14-P-HOME-7,8y].

### 3.2. Pragmatic Changes in Everyday Life

This category encompasses caregivers' experiences of pragmatic changes, regarding the modification of their daily life at economic and personal interests' level, as well as work situation.

#### 3.2.1. Work Situation and Economic Level

Caregivers had to reduce or interrupt their work for daily relative's assistance temporarily or definitively and it had a relevant impact on their income. A mother noted: “I have had to reduce my working time, I requested (paid,* authors' note*) parental leaves, so I cut my salary, because to do caring I used to leave work earlier” [4-M-HOME-7,4y]. Caregivers also spoke about worsened economic situation due to the costs for patient's care. For instance, one wife said: “Pharmacy costs cannot be quantified; however, I spend lot of money for him. I spend an average of € 1000 per month. You know, for example, I bought two pillows and I spent 250 Euros. It's quite expensive” [11-P-INST-3,6y].

#### 3.2.2. Daily Activities and Personal Interests

Caregivers' narratives included experiences of change in their previous personal life and interests and in how they used to spend their spare time. Their life is now focused on care activities, and leisure time is not the same as it used to be in the past. One mother said: “We used to go out for a pizza, you know. Now no. Nothing. No way” [12-M-HOME-5,7y]. This is an example of caregiver of patient living at home. Instead some differences emerged from caregivers of patients hosted in institutions. For example, a wife said: “I'm living in ‘another reality'. I have decided this: when I'm at (name of care institute,* authors' note*) with my husband it is one life, outside the gate it is another… I must remember I have grandchildren and children. During the day, my life is only with him, I must stay only with him. Then, out of there, I can feel free to go out for a dinner with my daughter or a friend of mine” [5-P-INST-2,2y].

### 3.3. Changes in Individual Perceptions

Participants spoke about changes in their perception of themselves (how they see themselves in terms of personality and roles) and how they perceive and define their relative with DOC.

#### 3.3.1. Personal Characteristics

Despite the difficult situation, many caregivers discovered themselves as being a new person with an unknown strength. For example, a mother said: “I have discovered to have an inner strength that I did not think to have, my self-esteem has increased, I was completely alone in a very difficult situation. I wouldn't expect, but I succeeded” [14-P-HOME-7,8y]. Instead, partners who have been taking care of their relative for less than two years, almost all had their relative in a care centre and did not speak explicitly about personal changes. However, from their narratives, it emerges that they had to change in order to manage their difficult situation. One partner said the following: “It was difficult for me, because I used to have always someone to help me doing everything, so it has been a great thing to find the courage to face problems and go ahead alone” (15-P-INST-1,9y).

#### 3.3.2. Caregiver's Role

The caregiver's role became a new and often predominant role. However the caregiver's role was not explicitly recognized by them, but it could be deducted from detailed descriptions of the activities directed to caring. A partner participant reported the following: “When it happened everything changed, my life has changed; now it is just to go to visit him, and that's all. I haven't had a pizza for 2 years leaving home only to go to visit him” [6-P-INST-4,7y].

#### 3.3.3. The Relative

Woman caregivers reported changes in how they see their relative. It is like she/he is another person with whom they shared their past. A mother said: “Everything you have built together remains, our bonds, and affection. You remember all things done with him, but the fact is that he is another person, someone to wash, dress and so on. So, I mean, it's like having another child, but with a past in common with me” [1-M-HOME-11y]. At a linguistic level when speaking about the relative caregivers confuse and simultaneously use present and past tenses. For example, a wife said: “Giovanni was… is an extraordinary person, he never argues with anyone” [9-P-INST-1,3y], and a mother said that “Sandro is a really handsome young man… he was really handsome” [2-M-HOME-2,7y].

### 3.4. Changes in Interpersonal Relationships

Changes also occurred in interpersonal relationships between caregiver, relative with DOC, and other significant persons. The interviewees described that they interacted with their relative with DOC in a different way, they developed some new methods of interaction, and they expressed feelings of nostalgia for their past relationship. They also changed their social relationships, cutting off past relations and building new ones.

#### 3.4.1. With the Relative in VS or MCS

The caregivers changed the way in which they see the relative and usually they describe their relative as another child but not as an adult (a partner or a grown up child). As a consequence, the way to interact with the relative changed, for example, simplifying their language and using expressions typical to children. This phenomenon regarded mother and partner, with no differences between places where the patients lived or time from the acute event. A partner said: “I see him as my fourth child. To me he's a little child, even when I talk to him. I call him ‘little one', ‘hi my little one', because he is like a helpless child” [5-P-INST-2,2y]. Furthermore, they missed their past relationship with the person in VS and MCS. A mother noted: “I really miss my daughter, because I don't know if she's still herself or if she's different. I had a very good relationship with her, we went out together, we chatted a lot, I miss her a lot” [13-M-HOME-0,9].

#### 3.4.2. With Significant Others

Caregivers experienced changes in their informal social networks of friends or relatives; in fact, a spouse reported the following: “You know, a lot of friends they disappeared, yes, but also my colleagues. I was expecting more attention and visits from them” [15-P-INST-1,9y]. A mother noted: “These situations make others run away, people run away. Sometimes people say to me ‘I can't come to see you'” [2-M-HOME-2,7y]. As the old network changed, some caregivers established new interpersonal relationships. Caregivers of patients at home established new relationships with other healthcare professionals coming daily or weekly to take care of the relative, while caregivers of patients in a care centre became friends with other caregivers with whom they daily shared their experience. A mother that had just come back home with her son said: “You know, the only positive side is that once you are admitted in the care centre you start to be part of a family, the other parents become your family” [4-M-HOME-7,4y].

### 3.5. Expressed Needs

The caregivers reported unmet needs for themselves. Interestingly, no different needs between mothers or partners were reported, while differences were mostly related to the place where patients live.

#### 3.5.1. Time for Doing Something for Themselves

Some needs were strongly related to time to do something for themselves and to take a break from the situation. This mainly concerned caregivers living with the relative with DOC. They reported the need to take “objective” time for themselves, due to the 24-hour-a-day assistance. A mother said: “I would like more time for my life, I'd like to ‘breathe' much more and, nowadays, I hardly have time to breathe because I'm handling too many things” [4-M-HOME-7,4y]. It is noteworthy that caregivers of patients at home, while saying that they had little time for themselves, reported that they felt they were unable to be away from their relative and they found it difficult to take “mental” time for a break from the situation. A mother said: “What I mostly miss is time for myself, I miss it because I'm busy, but also because, maybe, I wouldn't be able to take it, I wouldn't be able to take a break” [10-M-HOME-3.2y].

#### 3.5.2. Need for Simplified Pathways of Care

Caregivers of patients in a care centre reported a need to simplify their life and the management of their relative's health condition. A partner said: “What I need more than anything else is less bureaucracy, a straight way, so that one person starts, is admitted to a hospital and does everything in the same hospital” [8-P-INST-1y].

### 3.6. Perception about Future

Narratives included some considerations about the future. It seems that caregivers live in the present moment and refuse to think about the future.

#### 3.6.1. Living in the Present and Refusing to Think about the Future

Caregivers' life is focused on the present and future scares them. A partner said: “I think about tomorrow. About the future, never. Yes, the thought of saying ‘tomorrow' scares me. You know, if I didn't do like that I don't think I could live. Because I think about all the plans we made. One leaves home in the morning and then… all changes” [11-P-INST-3,6y].

#### 3.6.2. They Refuse to Think about the Relative's Future

They were also worried about who will take care of their relative when they will be no longer there. A mother described the following: “For example, about the future…when I think about the future I say, ‘Oh God, if something happens to me, what will become of him?' I delete, delete, delete…” [1-M-HOME-11y].

## 4. Discussion

The in-depth examination of interviews allowed us to detect six main themes regarding the changes after the acute event in women informal caregivers of patients in VS and MCS. All of them started telling their story from a general statement “Everything has changed, nothing is like before” and they reported some difficulties in integrating past, present, and future. They live in the present but long for the past, and they all say that future is unthinkable with the exception to very practical issues. This difficulty here defined as “time gap experience” is expressed in two ways. First, the reduction of variety of roles into one, caregiver's role, can be observed in different aspects of narratives, for example, when they speak about pragmatic changes in everyday life due to caring activities, reduction of other relationships, and difficulties in taking time for doing something for themselves. Second, the relationship with the relative is characterised by fluctuation in the relational style between caregiver and relative, it shifts from an adult to adult interaction to an adult to child one. Another fluctuation can be observed in the mixed use of present and past tenses when caregivers speak about their relative. These behaviours could be the expression of caregivers difficulties in understanding what person their relative became, frequently defined as another person with a shared past. The content of themes reported does not differ between the relationships between caregivers and the relative (mothers or partners) while differences for place of living (at home versus long-term care institutions) and time from acute event emerged.

As a consequence of becoming a caregiver, many things changed in their life. They have a new role added to the previous ones (e.g., a worker, a mother, a wife, etc.), but they do not explicitly recognize it. In fact, they do not refer to themselves as “caregivers” or persons that take care of someone. Activities of taking care of their relative fall under other roles. For example, “I am a mother, so I take care of him,” although being a mother is different from being a caregiver. As previously described [31] caregivers often do not identify themselves with a caregiver's role and report to lose their own identity [29]. Furthermore, authors described well how caregivers of patients with various diagnoses (Alzheimer's, Parkinson's, heart conditions, cancer, etc.) are unprepared for their caregiver's role, and they learn to be a caregiver from life experiences, health professionals, hospital workshops, or other sources, and they would like to receive different information at various times of the caregiving process [32]. In a recent study [17], more than 75% caregivers of patients in VS and MCS expressed not only a strong need for information about patient's clinical condition and treatment but also a need for learning how to take care of their relative. Therefore, to support caregivers of VS and MCS patients in their care activities, it could be important to provide some training in order to teach them how to manage care implications, to provide practical care for their relative, and to recognize and manage their caregiver's role and changes that occur in their life at a psychological level.

Our caregivers felt that their life completely changed and it is very different from the life they used to live. Authors have identified in caregivers of stroke survivors the experience of “losing the life that once was” [26]. At pragmatic level, caregivers had to reduce or interrupt temporally or definitively their work to have time to take care of the relative. Consequently, their economic status worsened because of the decrease of their income and the amount of care costs. They also changed their habits, hobbies and interests, and the way of spending their spare time [12, 13, 26, 37].

We observed differences related to the place of living: caregivers of patients in institutions have more possibilities to take a break from their caregiving activities, instead of caregivers of patients living at home who spend most of their time in caring activities. Caregivers show difficulties in adapting to the new situation that brings many important changes, among which changes in themselves and in their personal interests and hobbies. They also express a need for a simplification of patient's care pathway. White and colleagues [38] investigated factors facilitating the caregiver's role in caregivers of stroke patients. Among others, the coordination of care and the accessibility to community resources were considered facilitating factors while the lack of collaboration with the health care team and the lack of community support for the caregiving role represent barriers; see also [26]. Therefore, the coordination of services may help to alleviate caregiver burden [37], facilitating the assumption of caregiver's role.

Our caregivers also experienced changes in the previous informal social networks with the interruption of past relations (with relatives, friends, colleagues, etc.) but at the same time they constructed of new ones in particular with other caregivers of patients in VS and MCS. Caregivers of patients at home established new relationships with healthcare workers daily or weekly taking care of the patient; otherwise caregivers of patients in a care centre kept in touch with other caregivers attending the centre. These findings are supported by Huber and Kuehlmeyer [24] who explain new relationships by the fact that other caregivers of patients in VS may be able to understand their situation and they can share questions and problems that arise during the caregiving. Authors found that caregivers of patients in VS and MCS hosted in long-term care institutions reported problems in social involvement with the previous informal networks [12], and Chiambretto et al. [13] reported a general social restriction in caregivers of patients in VS. Our caregivers have difficulties in integrating past and present and also in relationships with members of their old and new social networks.

Ways in which caregivers spend their leisure time have changed too. In our research, caregivers expressed a need for leisure time for themselves. This need is also reported by Harding and Higginson [31] and the lack of time is often related to the considerable amount of time required for caregiving [12, 13]. In fact, it is important for caregivers to have some private space for themselves in order to relief the burden of care [39, 40]. At the same time, especially caregivers of patients living at home, while expressing need for more time for themselves, are unable to be away from their relative and have difficulties in taking a break from the situation. This is described also by Huber and Kuehlmeyer [24], “Family caregivers* (of patients in VS, authors' note)* need time for recreation but at first they might feel unable to be away from the patient” (p.104). So further research should be addressed to better understand this dilemma “I can not* versus* I do not want” to take a break from the patient; see also [30].

Considering the second aspect that explains the time gap experience, our caregivers have in front of them “a new person,” that at cognitive and physiological level is different from the person they used to know. As a consequence, based on their old and new perceptions about their relative they redefine him/her as a new person with a shared past [24]. Crawford and Beaumont [28] spoke about a paradoxical feeling because “the patient is no longer the person he previously was, although he might be seen as the same person.” It is interesting to note that at linguistic level, this phenomenon is expressed by a confusingly and simultaneously used present and past tenses when speaking about the relative (she/he is and she/he was). At interpersonal level, caregivers had to change the ways they interact with their relative. Due to their clinical condition, that requires constant care and limits substantially the interaction abilities, the patient is seen as a little child, and caregivers interact with him/her as if she/he was a little child. After the acute event, caregivers had to discover new ways to interact with their relative, because the previous ways did not work anymore. Saban and Hogan [26] identified a change in the caregivers' relationship with stroke patients: “The stroke survivors' personality was so drastically changed because of the stroke that the stroke survivor no longer seemed like the same person that the caregiver had known prior to the stroke.”

Finally, in addition to the difficulties in integrating present and past, caregivers have difficulties in integrating the future expectations to their present and past life. They refuse to think about their future and live fully involved in caring activities in the present or in the day after tomorrow but not any longer. This is probably due to the uncertainty of relative's clinical condition [26] and the fact that thinking about the future obliges them to think about the course of patient's condition and who will care of a patient when caregivers will not be there. Coherently, Chiambretto et al. [13] found that caregivers of patients in VS do not think about the possible death of their patient, while Huber and Kuehlmeyer [24] said “What is special to the caregiving for a patient in the VS is the uncertainty of the patient's mental capabilities and the patient's future recovery. It is not a condition that is necessarily deteriorating” (pg. 104).

Despite the difficult situation, our caregivers define themselves as a new person, discovering unknown inner strengths. These are mainly caregivers whose relative had an acute event within two years, probably because caregivers are still facing all changes that happened after the acute event that require many efforts and often bring caregivers to new insights or “discoveries” about themselves. Some caregivers learn to face the situation counting on their own strength and resources. Hamama-Raz et al. [25] described that wives of patients in VS experienced an empowered sense of self-esteem and inner strength related to their love and feelings of responsibility and commitment to their husband. At the same time, they reported a sense of isolation, emotional grief, reduction of hope and feelings of mourning.

Some limitations of this paper should be acknowledged. We recruited a convenience sample, caregivers came from the North of Italy. Thus, we think that it could be important to study caregivers' experience in other parts of Italy: we suppose that due to differences in pathways of care and economic conditions, it might vary. Furthermore, special attention should be given to the process during which caregivers shared their experience. It is important to acknowledge the role of the interviewer that is crucial in this process. The caregivers' stories have a very big emotional load and it would not be strange if they elicited some reactions of interviewers that would tend to rush through more sensitive themes instead of encouraging the caregiver to share his/her story to the extent where he/she still feels comfortable. We were aware of this risk and we hope that it was at least in great part overcome, because the interviewers were experienced and also they reflected on the data collection process.

## 5. Conclusion

Overall, informal caregivers indicated many personal and interpersonal changes and some difficulties in integrating their past, present, and future life, as if they were suspended in the passing time. From the acute event, caregivers' life has changed and it seems as if they fluctuate from the present to the past, to which they are intrinsically linked. It is like they are suspended in a today, holding to the past, and not linking to the possible future, that is what we define the “time gap experience.” This difficulty might be related to the uncertainty of patient's clinical condition and also its course. This phenomenon is inferred from how caregivers describe themselves and their relative at linguistic level and how they think about the future. Further research could be directed to better understand the psychological mechanism responsible for the time gap experience considering the possible influence of fluctuation between past-present-future on caregivers' burden. A better understanding about changes in caregivers' role and relationships in caregivers' life might support targeted interventions aiming to empower their capabilities and personal new abilities and to support a better integration between past and present may be useful to promote and improve their health and quality of life for both patient and caregiver.

## Figures and Tables

**Table 1:** Demographic data on caregivers and their relatives.

Demographic data	*N* (%)
*Caregivers *	
Age	
Mean 57 (range: 32–78 years)	
Under 40	1 (6,7)
Between 41 and 60	8 (53,3)
Between 61 and 80	6 (40,0)
Education levels	
Primary school	3 (20)
Secondary school	3 (20)
High school	8 (53,3)
Graduate degree	1 (6,7)
Resignation from job	
Yes	7 (46,7)
No	8 (53,3)
Relationship with patient	
Spouse/partner	7 (46,7)
Mother	8 (53,3)
*Patients *	
Age	
Caregivers' child: Mean 33 (range 20–47)	
Caregivers' partner: Mean 57 (range 33–68)	
Diagnosis	
Vegetative state	7 (46,7)
Minimally conscious state	8 (53,3)
Place where patient is hosted	
At home	8 (53,3)
Postacute rehabilitation care	0
Long-term care	7 (46,7)
Duration of patient health condition	
<2	5 (33,3)
2–5	5 (33,3)
5–10	4 (26,7)
>10	1 (6,7)

**Table 2:** Details on caregivers interviewed in chronological order.

Interview number	Relationship with patient	Patient's diagnosis	Place where patient is hosted	Duration of patient health condition (years)
1	Mother	MCS	At home	>10
2	Mother	MCS	At home	2,1–5
3	Mother	VS	Long-term care	2,1–5
4	Mother	VS	At home	5,1–10
5	Spouse	VS	Long-term care	2,1–5
6	Spouse	MCS	Long-term care	2,1–5
7	Mother	MCS	At home	>10
8	Spouse	VS	Long-term care	<2
9	Spouse	VS	Long-term care	<2
10	Mother	MCS	At home	2,1–5
11	Spouse	MCS	Long-term care	2,1–5
12	Mother	VS	At home	5,1–10
13	Mother	VS	At home	<2
14	Spouse	MCS	At home	5,1–10
15	Spouse	MCS	Long-term care	<2
